# Characterization of an air-liquid interface primary human vaginal epithelium to study Ebola virus infection and testing of antivirals

**DOI:** 10.1016/j.antiviral.2023.105551

**Published:** 2023-01-31

**Authors:** Olivier Escaffre, Vsevolod Popov, Eldridge Hager, Alexander N. Freiberg

**Affiliations:** a Department of Pathology, USA; b Center for Biodefense and Emerging Infectious Diseases, USA; c Institute for Human Infections & Immunity and Sealy & Smith Foundation, University of Texas Medical Branch, Galveston, TX, 77555, USA

**Keywords:** Human vagina, Ebola virus, Sexual transmission, Polyphenylene carboxymethylene

## Abstract

Ebola virus (EBOV) is the causative agent of the often-fatal Ebola virus disease (EVD) characterized by hemorrhagic fever in humans and non-human primates. Sexual transmission from male survivors has been at the origin of multiple outbreak flare-ups between 2015 and 2021. However, this route is still poorly understood and the resulting EVD from it is also understudied. To support epidemiological studies documenting sexual transmission to women, and as a transition from previously using monolayer vaginal epithelial cells (VK2/E6E7), we first determined the biological relevance of two similar air-liquid interface models of the human vaginal epithelium (VEC and VLC Epivaginal^™^) and then characterized their susceptibility to EBOV and virus-induced inflammation. Finally, we evaluated toxicity of Polyphenylene Carboxymethylene (PPCM) microbicide in VLC and reassessed its antiviral effect.

As expected, the VEC, but also VLC model showed stratified layers including a lamina propria under an epithelial structure similar to the full thickness of the human vaginal epithelium. However, we could not detect the immune cells featured in the most relevant model (VLC) of the vaginal epithelium using the dendritic cell CD1a and CD11c markers. Consistent with our previous work using the VK2/E6E7 cell line, infectious virus was detected from the apical side of both primary human cell systems, but only when using a high infective dose, with titers remaining at a constant level of 10^3–4^ pfu/ml over 7 days suggesting lasting infectious virus shedding. In addition, infection caused disruption of the epithelium of both models and virus antigen was found from the apical superficial layers down to the lamina propria suggesting full virus penetration and overall confirming the susceptibility of the human vaginal tissue for EBOV. Just like previously seen in VK2/E6E7 cells, VLC infection also caused significant increase in inflammatory markers including IL-6, IL-8, and IP-10 suggesting vaginitis which is again consistent with tissue lesions seen in non-human primates. Finally, both virus infection and virus-induced inflammatory response in VLC could be prevented by a single 5-min PPCM microbicide treatment prior infection.

## 1. Introduction

Ebola virus disease (EVD) is characterized in humans by hemorrhagic fever with high mortality rates (Feldmann et al., 2013; Feldmann and Geisbert, 2011). EVD is caused by Ebola virus (EBOV), a member of the *Filoviridae* family, which in addition to causing acute disease, can also persist in the body of survivors at immunologically protected sites, such as the brain, eyes, or testes (Glynn et al., 2017; Jacobs et al., 2016; Kelly et al., 2022; Sow et al., 2016; Varkey et al., 2015). Sexual transmission of filoviruses is now well recognized while it was first suspected with Marburg virus more than fifty years ago (Martini and Schmidt, 1968; Slenczka and Klenk, 2007). While EBOV has never been demonstrated in humans, it is likely to be present in the testes, because viral genome has been found in the semen of many survivors during the 1995, 2000, and 2014–2016 outbreaks in West Africa (Sow et al., 2016; Rodriguez et al., 1995; Sissoko et al., 2017; Barnes et al., 2017; Deen et al., 2017; Fischer et al., 2017; Keita et al., 2017; Emond et al., 1977; Rowe et al., 1999; Subtil et al., 2017; Uyeki et al., 2016). As a result, EBOV was effectively transmitted and caused death in multiple women (Thorson et al., 2016; Mate et al., 2015; Diallo et al., 2016; Den Boon et al., 2019) suggesting that EBOV virulence following sexual transmission is not attenuated when compared to the disease contracted through other routes. In fact, sexual transmission was considered non-insignificant as it caused several outbreak flare-ups (Den Boon et al., 2019; Subissi et al., 2018) and isolation of infectious individuals plus abstinence could eradicate the disease (Luo et al., 2019). Despite the availability of vaccines, since late 2019 (Tomori and Kolawole, 2021) several EBOV outbreaks, mostly from spillover events, have been recorded in the Democratic Republic of the Congo (n = 4) and in Guinea (n = 1) (April 2022). Some of these cases have again emerged because of either sexual transmission of a survivor or relapse (CDC, 2020; CDC, 2021). Regarding laboratory experiments, there is still no published in vivo study demonstrating sexual transmission. However, several studies in macaques and guinea pigs have found virus genome or antigen in tissues, and glands of the male but also female genital system following an intra-muscular or peritoneal challenge (Cooper et al., 2018; Liu et al., 2022; Perry et al., 2018; Baskerville et al., 1985; Johnson et al., 2021; Zeng et al., 2017). This suggests that both genders can be infectious and ultimately infect their partners by the sexual route. These data are also supported by the fact that undifferentiated human VK2/E6E7 cells, originally isolated from a normal vaginal mucosal tissue, are permissive to EBOV (Escaffre et al., 2019). Altogether, these studies highlight the need for characterizing infection of the reproductive system in women and for development of antiviral medical countermeasures that specifically limit sexual transmission of EBOV and prevent outbreak flare ups.

Microbicides are an adequate choice to face this route of transmission when administered through a topical gel to the vaginal mucosa prior potential exposure. These compounds are selected for their broad-spectrum activity against sexually transmitted infections of viral and bacterial origin and have the advantage of being widely distributed and available at low cost. To this date, there is no data about testing of microbicides against EBOV in the context of a sexual transmission other than demonstrating polyphenylene carboxymethylene (PPCM) efficacy using monolayer vaginal and cervical epithelial cells (Escaffre et al., 2019). PPCM is a multipurpose prevention technology drug designed to protect women from unplanned pregnancy and disease (Chang et al., 2007; Herold et al., 2002; Zaneveld et al., 2002). Specifically, it has been shown to be effective at blocking EBOV, HIV, herpesvirus, and bacterial infections in vitro or in vivo (Escaffre et al., 2019; Chang et al., 2007; Herold et al., 2002; Zaneveld et al., 2002). PPCM also showed in vitro protection against respiratory pathogens including SARS-CoV-2 (Escaffre and Freiberg, 2021). While the mechanism of action of PPCM against EBOV needs to be further investigated, it was shown to individually bind to the virus glycoprotein and cells (Escaffre et al., 2019), possibly through the glycan cap in the mucin-like domain of GP1, and the cell surface glycosaminoglycan heparan sulfate, respectively.

To transition from our previous work studying EBOV in a two-dimensional vaginal epithelium model (VK2/E6E7 cells) (Escaffre et al., 2019) and prior animal testing, we further characterized the infection in air-liquid interface three-dimensional models (VEC and VLC EpiVaginal^™^) mimicking the stratified vaginal epithelium. In addition, these biologically relevant primary models of the vaginal epithelium were used to confirm PPCM antiviral properties against EBOV. VEC is a well-accepted in vitro model of the vaginal epithelium (Ayehunie et al., 2006, 2011, 2015) and both VEC and VLC offer a good alternative to using animal models and for pre-clinical testing (Ayehunie et al., 2006, 2011, 2018). Our results confirm that both models structurally resembled the human vaginal epithelium and demonstrate their permissiveness to EBOV including cells of the *stratum basale* and lamina propria which is suggestive of virus dissemination into the reproductive system. We also show that VLC mounted a robust immune response to infection and that both virus infection and secretion of proinflammatory molecules could be significantly reduced by a single PPCM treatment prior infection.

## 2. Material and methods

### 2.1. Cells, chemicals, and virus

VEC and VLC EpiVaginal^™^ models were purchased from Mattek corporation and cultured on inserts (0.4 μm pore size) at air-liquid interface as previously described (Ayehunie et al., 2006, 2015, 2018). Both vaginal epithelial models originated from the ectocervix collected from a healthy single donor undergoing hysterectomy. The ectocervix is part of the vaginal wall and was used as the source of normal fibroblasts and vaginal epithelial cells. Fibroblasts were seeded onto the lamina propria equivalent on top of which vaginal epithelial cells were further cultured and differentiated for 14 days to give a multilayered epithelium with a non-cornified vaginal phenotype (Ayehunie et al., 2015, 2018). Note that the VLC model features immunocompetent dendritic cells that were inserted into the epithelial layers and lamina propria equivalent. Once received, VEC and VLC cultures were used at 37 °C, 5% CO_2_ in a 6-well plate format on double washer stands in presence of 5 ml per well of serum-free Dulbecco’s Modified Eagle’s Medium/F12 culture medium supplemented with the epidermal growth factor (other proprietary factors included), gentamicin (5 μg/ml), and amphotericin B (0.25 μg/ml). Note that 2.5 ml per well of fresh culture medium was added every day to allow for sample collection during virus replication kinetics. Vero-E6 (CRL-1586), THP-1 (TIB-202^™^), and Jurkat (Clone E6–1, TIB-152) cells were purchased from the American Type Culture Collection (ATCC) and maintained as instructed by the manufacturer.

Polyphenylene carboxymethylene (PPCM) polymer was provided by Yaso Therapeutics Inc. PPCM stock solution was prepared in phosphate-buffered saline (PBS) at 220 mg/ml and sterile-filtered at 0.2 μm prior use. A 4% PPCM solution was used in this study to be consistent with previous toxicity studies performed in rabbits and rats (Zaneveld et al., 2002). PPCM cell toxicity after 12 h contact with VLC and VEC apical side was carried out by standard MTT and LDH assay (Sigma-Aldrich). Note that samples for LDH activity measurement were collected from the basal side of cultures.

Wild-type Zaire Ebola virus (Mayinga isolate) was propagated in mycoplasma-free Vero E6 cells. Virus titers were quantified by plaque assay technique using Vero E6 cells, as previously described (Escaffre et al., 2019) and expressed as log_10_ (pfu/ml). Note that samples were also plaqued undiluted (150 μl inoculum) to increase sensitivity. Infectious work was performed in a class II biological safety cabinet in the biosafety level 4 laboratory (BSL4) of the Galveston National Laboratory at the University of Texas Medical Branch (UTMB).

### 2.2. Trans-epithelial electrical resistance (TEER)

TEER of VEC and VLC was evaluated during PPCM cell toxicity assay and virus replication kinetics using the EVOM^2^ epithelial voltohmmeter system (WPI) and the Endohm-12G chamber. Clearance between top electrode and culture membrane was adjusted to 2 mm. Each insert (mock first) was placed in the chamber with 200 μl and 900 μl of culture medium in respectively the apical or basal side, and resistance in ohm was recorded.

### 2.3. Cell infection

Prior to virus inoculation, inserts were washed on the apical side with 250 μl of 1x phosphate-buffered saline (PBS) to remove any cell debris. Infection was performed with 100 μl virus at a multiplicity of infection (MOI) of 5 and 0.01. The MOI was calculated based on an estimation of the cell number used to reach 100% confluency at the time of seeding an insert. Virus contact was allowed for 1 or 12 h at 37 °C. Note that the virus wash with 150 μl 1X PBS was only performed for inserts undergoing 1 h infection. All liquids were then removed from the surface to maintain the differentiation process. Where appropriate, PPCM treatment was applied to the apical side of inserts 5min prior addition of virus for 12 h. Each condition was performed with biological duplicates or triplicates. Sampling during the initial virus replication kinetic (1 h virus contact) was performed from the apical side by washing the cells daily with 250 μl of 1X PBS and collecting 250 μl of culture medium from the basal side. Sampling during the virus kinetic assessing PPCM efficacy was done immediately after removing the 12 h virus inoculum, and then daily starting 2 days post infection.

### 2.4. Electron microscopy

Mock-infected VLC inserts were fixed for scanning electron microscopy technique as previously described (Escaffre et al., 2016). Briefly, inserts were fixed for 72 h in 2.5% formaldehyde and 0.1% glutaraldehyde in 0.05 M cacodylate buffer (0.01% trinitrophenol, 0.03% CaCl_2_, pH 7.2). Samples were then washed, dehydrated in ethanol, and processed through hexamethyldisilazane. After being air-dried filters were detached, mounted onto the specimen stubs and sputter-coated with iridium in Emitech K575x sputter coater (Ashford, Kent, England) at 20 mA for 20 s. Other inserts were also similarly processed until the washing step for transmission electron microscopy purpose. Samples were then incubated in 2% aqueous uranylacetate, dehydrated, infiltrated with Poly/Bed 812 prior embedding into polyethylene capsules for polymerization and sample sectioning.

### 2.5. Bright field and fluorescence microscopy

Inserts were fixed in 1:10 buffered formalin for 48 h, and then for 24 h with fresh formalin before removal from BSL4 for processing and paraffin embedding. Sections stained by hematoxylin, and eosin (H&E) were imaged with an Evos XL core. Cells and sections stained for CD1a, CD11c, and virus antigen were imaged with an Olympus IS71 inverted fluorescence microscope. CD1a, CD11c, and EBOV glycoprotein were detected using respectively a mouse anti-CD1a (abcam 238,463; dilution: 1/100), -CD11c (abcam 52,632; dilution: 1/100), or a rabbit anti-EBOV GP pAb (IBT0301–015; dilution: 1/200) coupled to either a goat anti-rabbit AF594 or anti-mouse AF488 (Invitrogen; dilution: 1/200) as secondary antibody. DAPI staining (Sigma) was added to localize round cell nuclei and visualize the whole structure.

### 2.6. Cell stimulation assay

Cell stimulation was done by incubating for 48 h 5 × 10^6^ Jurkat or macrophage-derived THP-1 (resting M0) cells with basal medium, or medium samples collected from the basal side of VLC cultures at day 6–7 post-infection. Note that cell viability was assessed by trypan blue counterstaining using Cell countess 3 (Invitrogen) and was above 80% at the time of stimulation. Positive controls of cell activation were also included and generated as following. Jurkat cells activation was done by 48 h of phytohemagglutinin (Sigma, 10 μg/ml) and phorbol 12-myristate 13-acetate (Sigma–Aldrich, 20 ng/ml) stimulation. Differentiation of THP-1 into resting macrophage (M0) was done by incubation with phorbol 12-myristate 13-acetate (185 ng/ml, Sigma–Aldrich) for 24 h. Polarization of M0 into a macrophage with a pro-inflammatory phenotype (M1 positive control) was done by 48 h of INF-γ (20 ng/ml, Immunotools), LPS (100 ng/ml, Sigma–Aldrich), and phorbol 12-myristate 13-acetate (Sigma–Aldrich, 20 ng/ml) stimulation. Polarization of M0 into a macrophage with an anti-inflammatory phenotype (M2a positive control) was done by 48 h of IL-4 (20 ng/ml, Immunotools), IL-13 (20 ng/ml, Immunotools), and phorbol 12-myristate 13-acetate (Sigma–Aldrich, 20 ng/ml) stimulation.

### 2.7. Flow cytometry

Jurkat T cells and macrophage derived THP1 cells were labeled with directly conjugated antibodies for specific activation surface markers. Specifically, Jurkat T cells were labeled with CD25 BV421 and CD69 PE antibodies (BD Biosciences). Macrophage-derived THP1 were labeled with CD80 BV421 and CD86 PE antibodies (BD Biosciences). Samples were then fixed in 4% paraformaldehyde for 30min prior PBS washing and acquisition on a BD LSR II (Fortessa) flow cytometer (BD Biosciences). A total of 50,000 to 100,000 gated events were collected. Analysis of data was performed by BD FACSDiva^™^ and FlowJo v10 software (BD Biosciences). Unstained controls and isotype controls that matched antibodies labeled with the same fluorochromes were used to determine background, gate positive cells, and detect changes in expression of surface markers.

### 2.8. Bio-plex assay

Culture medium from the basal side of VLC inserts was collected and inactivated on dry ice by gamma irradiation (5 Mrad). Analysis of inflammatory markers was performed at BSL2 using a Bio-Plex Pro Human cytokine group 1 panel 27-plex kit (Bio-Rad), as previously described (Escaffre et al., 2019). Quantification of 27 analytes was performed (Eotaxin, FGF-basic, G-CSF, GM-CSF, IL-1Rα, IL-1β, IL-2, IL-4, IL-5, IL-6, IL-7, IL-8, IL-9, IL-10, IL-12 (p70), IL-13, IL-15, IL-17, IFN-γ, IP-10, MCP-1, MIP-1α, MIP-1β, RANTES, TNF-α, PDGF-BB, and VEGF).

### 2.9. Statistical analyses

Comparison of virus replication in VEC and VLC at any given time was performed with a T-test. The significance of changes seen in TEER, viability, toxicity, expression of markers, or quantity of inflammatory molecules following various treatments was assessed by One-way ANOVA followed by Tukey’s multiple-comparison test (* p < 0.05, ** p < 0.01, *** p < 0.001, **** p < 0.0001). Results are presented as mean with standard deviation. Each condition was tested with 2–3 biological replicates due to the limited number of inserts.

## 3. Results

### 3.1. The EpiVaginal tissues mimic the human vaginal epithelium

**Fig. 1.**
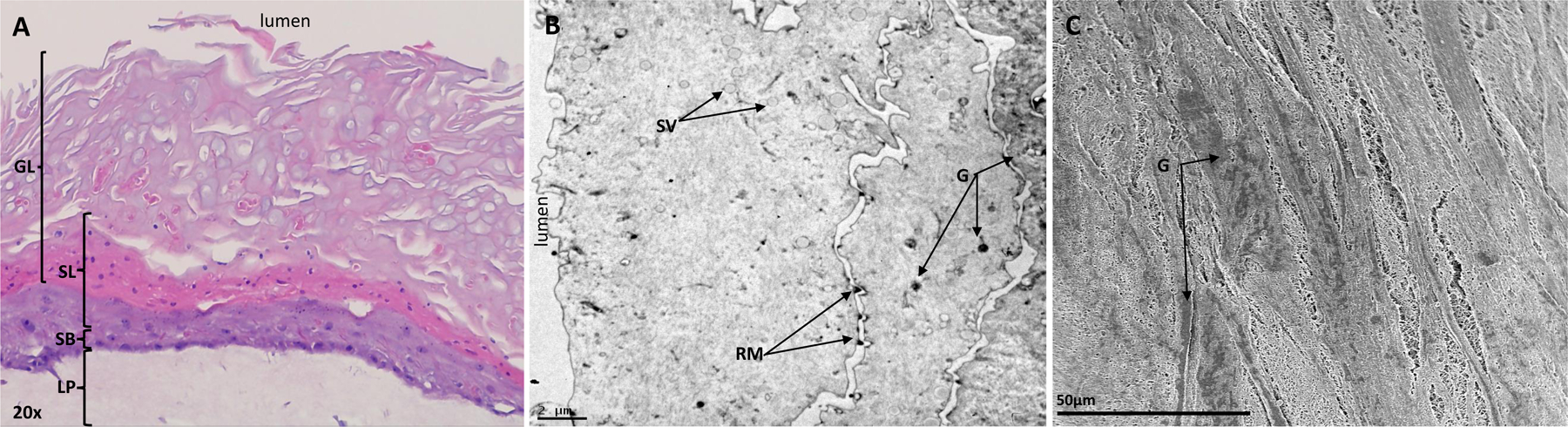
Imaging of VLC epithelium. Representative cross section of VLC (**A**) resembling the human stratified squamous vaginal epithelium and is composed of a lamina propria (LP), a *stratum basale* (SB), a suprabasal layer (SL), and superficial glycogen layers (GL). 20× magnification. Transmission electron microscopy of the apical surface of VLC (**B**) showing epithelial cells lacking nuclei, and cytoplasmic organelles. Secretory vesicles (SV), residual microvilli, and glycogen (G) can be observed. Scale bar: 2 μm. Scanning electron microscopy of the apical surface of VLC (**C**) showing undifferentiated cells with glycogen deposits (G). Scale bar: 50 μm. Note that inserts were fixed at the day 7 time point.

VEC and VLC models were first processed for imaging to confirm their structural resemblance with the human vaginal epithelium and suitability to study sexual transmission in women. Observation of H&E-stained VLC cross sections showed a multilayer model that included a lamina propria with elongated fibroblast-like cells and an epithelial structure made of a basal layer, several non-cornified layers, and finally apical layers of loosely connected epithelial cells (Fig. 1A). Specifically, the VLC epithelial structure showed the nucleated basal layer called *stratum basale*, and several suprabasal nucleated cell layers where glycogen was abundant. This was followed by multiple layers in which glycogen was still found but fewer nuclei were present (Fig. 1A), which is reminiscent of some superficial apical layers and where terminally differentiated epithelial cells are normally present to form the *stratum corneum* (Anderson et al., 2014). Observations by transmission electron microscopy confirmed that some epithelial cells at the apical surface of VLC were devoid of nuclei and most of cytoplasmic organelles and contained glycogen rosettes (putative) (Fig. 1B). However, observations by scanning electron microscopy did not show typical polygonal flaky corneocytes, microfolds, or elongated microvilli (Anderson et al., 2014) that define the *stratum corneum* but rather flat undifferentiated cells where glycogen deposits could be observed (Fig. 1C). Note that similar observations were found using the VEC model (data not shown). Overall, our data suggest that both VEC and VLC have structures that are reminiscent of the lamina propria, *stratum basale*, the suprabasal layers and other upper layers of the human stratified vaginal epithelium below terminally differentiated corneocytes. Finally, immunofluorescence imaging of VLC sections for CD1a and CD11c markers was done to stain for resident dendritic cells (Pena-Cruz et al., 2018; Wu et al., 2018; Zhao et al., 2003) that are included in this model as they constitute early and important EBOV targets (Lubaki et al., 2013). Unfortunately, while these markers were expressed on either THP-1 or Jurkat control cells (Supplementary Figs. 1A and B), no specific signal was observed in any of the VLC cross sections (Supplementary Fig. 1C) suggesting that dendritic cells were absent. However, other dendritic cell markers should be used to confirm these results, but this will require further investigations.

### 3.2. VEC and VLC are susceptible and permissive to EBOV

To transition from our previous work using two-dimensional undifferentiated culture systems, a high and low multiplicity of infection (MOI of 5 and 0.01, respectively) was applied for 1 h on the apical side of VEC and VLC to investigate their susceptibility and permissiveness to the virus. Both TEER measurement and sampling from the apical and basal side of each culture were performed daily for 7 days to monitor for active replication, and virus invasion through the models. Surprisingly, TEER rapidly and similarly decreased in mock- and virus-infected VEC and VLC tissues over the course of infection and by on average 75% by day 7, when compared to their corresponding resistance values at time of reception (Supplementary Figs. 2A and B), suggesting significant non-virus-related changes in epithelium barrier integrity. This was confirmed by visual inspection of inserts that all showed tissue retraction from the outer edge, as seen with VLC at day 7 post infection (Supplementary Fig. 2C). While this observation did not allow us to ultimately measure potential permeability changes caused by EBOV, we proceeded with the study as VEC and VLC tissues, that are more biologically relevant than a two-dimensional model, remained firmly attached to the membranes and their structural analysis reported to be as expected until the end of the study.

**Fig. 2.**
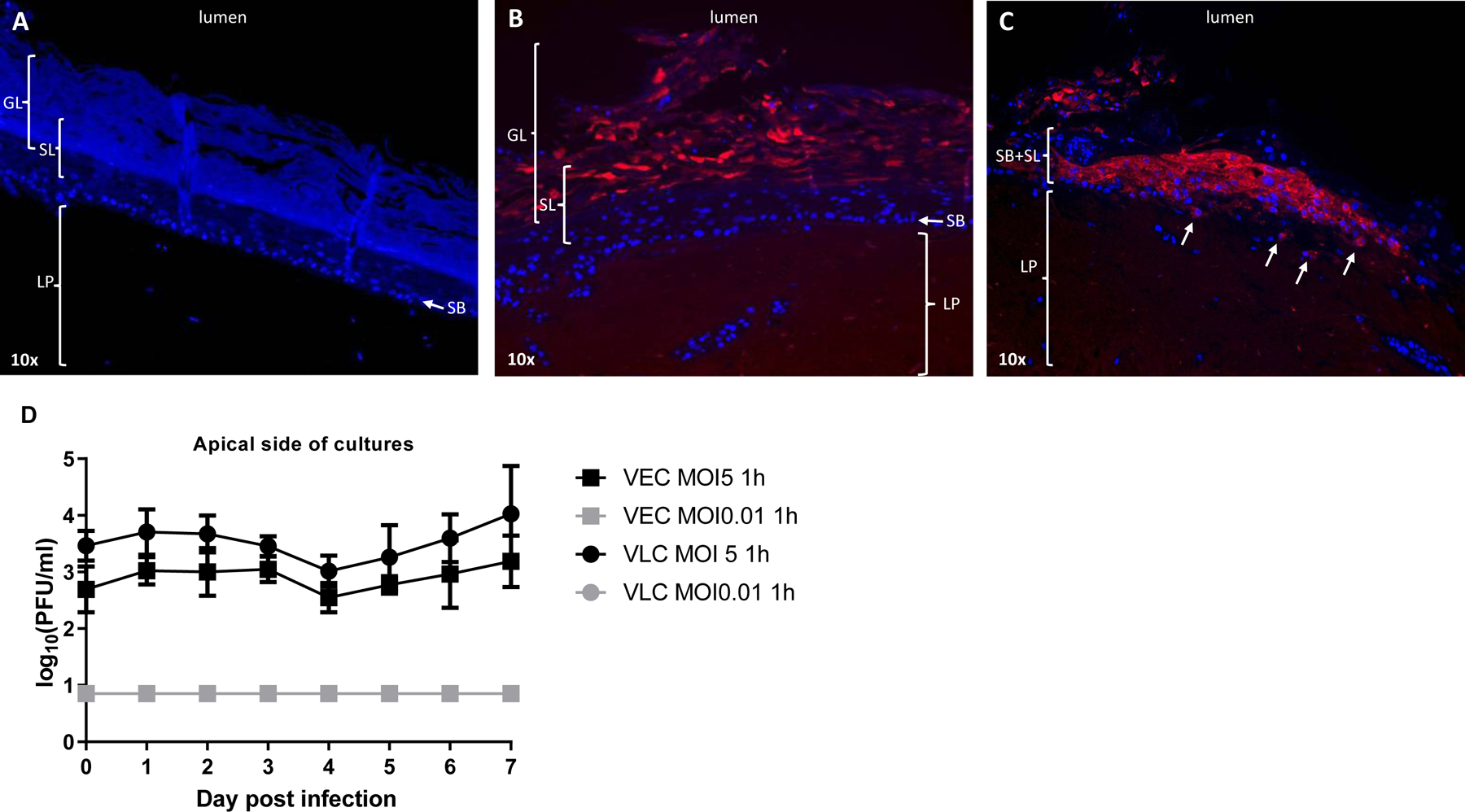
EBOV tropism and replication in VLC and VEC at a low and high infective dose. Representative VLC cross sections (**A**, **B**, **C**) showing EBOV-infected epithelial cells at day 7 post infection. Mock-infected section (**A**) showing the lamina propria (LP), the *stratum basale* (SB), the suprabasal layer (SL), and superficial glycogen layers (GL). DAPI staining (blue) was used to show the overall section structure. EBOV-infected sections (**B**, **C**) showing virus glycoprotein (red) in the superficial epithelial layers (**B**) as well as in the suprabasal layer, *stratum basale* and lamina propria (**C**). White arrows show virus antigen in cells of the lamina propria (**C**). 10× magnification. Virus replication kinetic (**D**) in VEC (square shape) and VLC (round shape) over 7 days. Kinetic data are from using a high or low multiplicity of infection (MOI) and are expressed as the average of 2–3 biological replicates with error bars showing standard deviations. Limit of detection (LOD) set at 10°^.85^ pfu/ml. Unpaired T-test did not show significant differences in terms of virus replication between VEC and VLC at comparable MOI.

Immunofluorescence imaging of high MOI virus-infected VLC showed by day 7 presence of EBOV glycoprotein (GP) throughout the entire stratified structure from the glycogen filled layers down to the lamina propria (Fig. 2A, B, C). Interestingly, GP staining in the lowest suprabasal layers, basal layer, and lamina propria was always observed when most of the upper glycogen filled layers were missing or severely disrupted (Fig. 2C). Similar observations were made using a high infective dose in the VEC model (data not shown), suggesting that both models are susceptible to infection, just like undifferentiated vaginal epithelial cells (VK2/E6E7), and that EBOV causes cytopathic lesions throughout the whole vaginal epithelial structure.

Sample titration showed that infectious EBOV could be detected from the apical side of both models for at least 7 days following a high MOI challenge (Fig. 2D). The amount of infectious virus between VEC and VLC were comparable (p > 0.05) and remained similar throughout the kinetics as they only increased by about 1 log_10_ and peaked at day 7 to an average of 10^3.5^ PFU/ml. Conversely, no infectious virus could be detected throughout the study (LOD of 10°^.85^ PFU/ml) when a low infective dose was used (Fig. 2D), although GP staining was observed in the suprabasal layers and higher at day 7 post infection in both models (data not shown). While these results support the fact that there are no intrinsic differences between VEC and VLC regarding their permissiveness to EBOV they also suggest that virus can persist in the vaginal epithelium to some levels and remain infectious for at least a week when isolated from the vaginal canal. Note that no infectious virus was retrieved from the basal chamber of an empty insert inoculated with a high dose of EBOV for 12 h, and as expected, from any samples collected from the basal chambers of both models (data not shown) indicating that virus dissemination can only be inferred from the immunostaining work when using these models.

### 3.3. Polyphenylene carboxymethylene (PPCM) pretreatment decreases EBOV infection of VEC and VLC

**Fig. 3.**
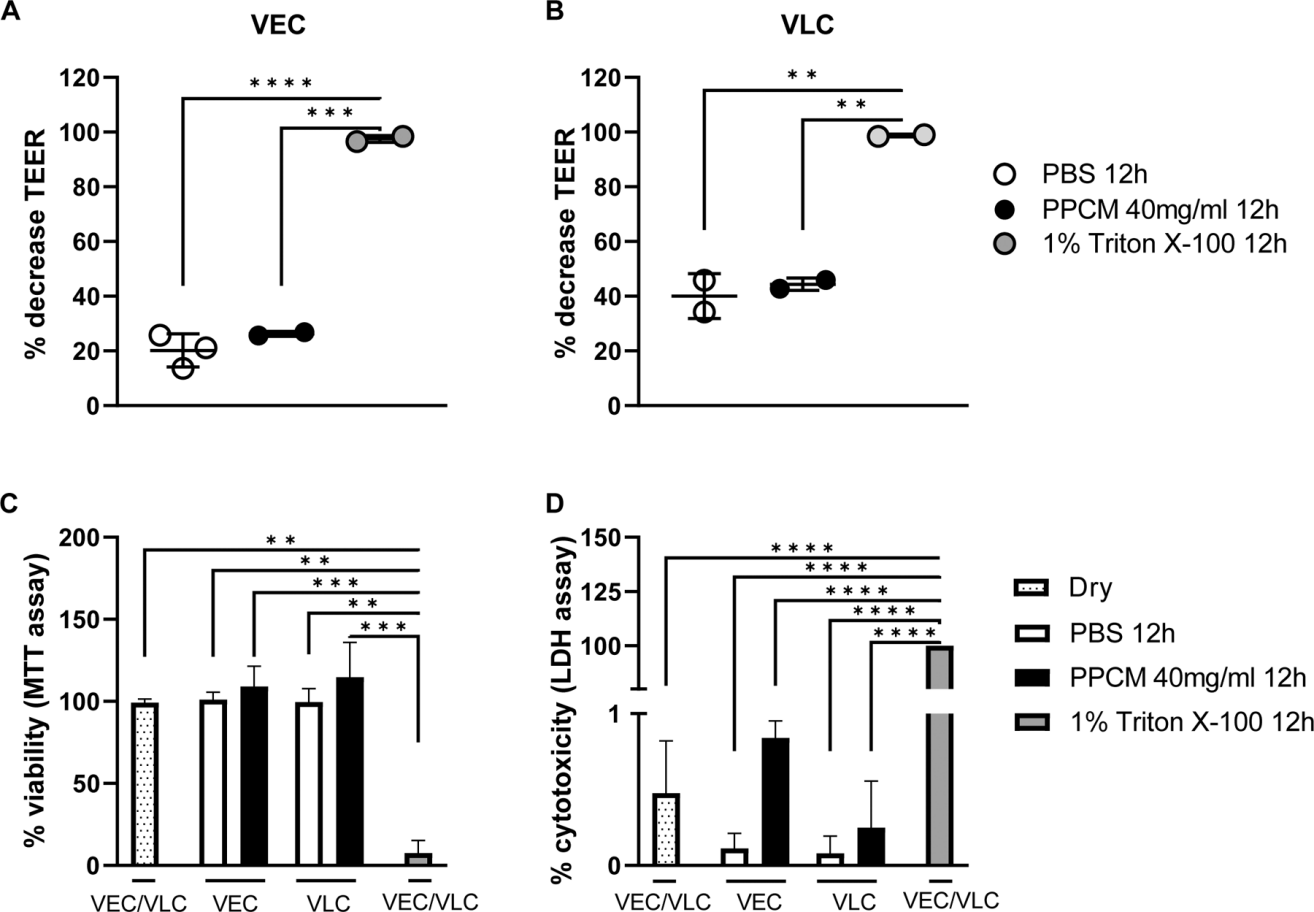
PPCM safety profile in VEC and VLC. Changes in trans-epithelial electrical resistance (TEER) in VEC (**A**) and VLC (**B**) following PBS, PPCM, or Triton X-100 treatment for 12 h. PPCM toxicity in VEC and VLC as measured by MTT (**C**) and LDH (**D**) assay following the same treatments. Results are expressed as the average of 2–3 biological replicates with error bars representing standard deviations. ** p < 0.01, *** p < 0.001, **** p < 0.0001 (ANOVA-Tukey’s multiple-comparison test).

This study also builds on previous experiments demonstrating antiviral activity of PPCM at 500 μg/ml or lower against EBOV in submerged two-dimensional cultures of undifferentiated human cervical (HeLa) and vaginal epithelial (VK2/E6E7) cells (Escaffre et al., 2019). However, PPCM was here used at 40 mg/ml to match the concentration previously used for vaginal toxicity studies (Zaneveld et al., 2002) since these models resembles the normal human vaginal epithelium. TEER of PBS-treated VEC and VLC decreased by respectively 20.2% and 44.4% after only 12 h of culture when compared to their initial corresponding values (Fig. 3A and B), which is again consistent with tissue retraction. These changes were also observed to the same extent in PPCM-treated VEC and VLC (p > 0.05) but were distinct, as expected, from those of the 1% Triton X-100-treated controls (p < 0.01) showing on average a 98.1% decrease during the same timeframe (Fig. 3A and B). Consistent with TEER data, cellular metabolic activity and plasma membrane integrity levels following PBS and 40 mg/ml PPCM treatment in VLC or VEC remained similar to those from dry insert controls (p < 0.05) (Fig. 3C and D). Note that higher PPCM concentrations were also tested in both models, and a CC_50_ higher than 190 mg/ml was determined (data not shown). As expected, 1% Triton X-100 control treatment dramatically reduced cell viability and cell integrity in both models, when compared to their counterparts from dry, PBS-, or PPCM-treated inserts (p < 0.01) (Fig. 3C and D). Altogether, these data suggest that 40 mg/ml PPCM was not cytotoxic in either model.

**Fig. 4.**
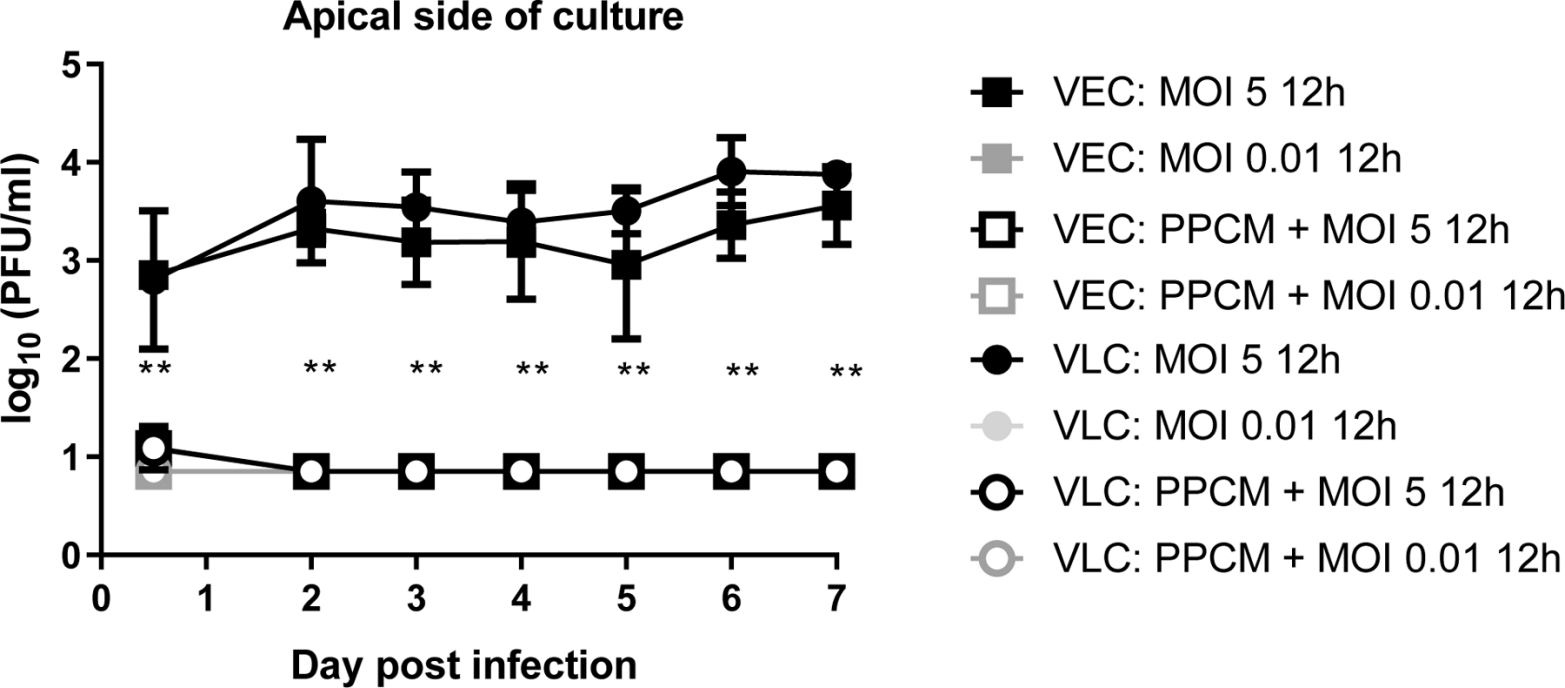
PPCM antiviral efficacy against EBOV in VEC and VLC. EBOV replication kinetic in VEC and VLC over 7 days. Assay performed using an initial high and low MOI for 12 h. PPCM treatments started 5 min prior infection (pre-treatment) where appropriate. Limit of detection (LOD) set at 10°^.85^ pfu/ml. Results are expressed as the average of 2–3 biological replicates with error bars representing standard deviations. Statistical differences in terms of virus replication are shown between PPCM- and non-treated samples using a high MOI of 5. ** p < 0.01 (Unpaired T-test).

PPCM is intended to be used prior intercourse and thus was applied to the apical side of VEC and VLC for 5 min as a one-time treatment. Virus was then directly added (MOI 5 and MOI 0.01) onto the inserts for 12 h before removing the inoculum and do the first sample collection. Consistent with the 1 h virus contact kinetic data, Infectious virus was only detected when a high infective dose was used (LOD of 10°^.85^ PFU/ml), regardless of the time point and culture model (Fig. 4). The detectable amount of virus also plateaued over 7 days as it only increased at best by 1 log_10_ and peaked this time to an average of 10^3.75^ PFU/ml (Fig. 4). Interestingly, PPCM treatment significantly decreased virus infection from both VEC and VLC for at least 7 days (p < 0.05) when a high infective dose was initially used (Fig. 4). Specifically, differences of virus titers at 12 h post infection were of 1.8 and 1.7 log_10_ between vehicle and PPCM-treated infected VEC or VLC cultures, respectively. Virus titer in all PPCM-treated cultures was then below the limit of detection between day 2 and 7 post infection, which was at least a 2.9 log_10_ decrease (p < 0.01). As expected, no infectious virus was retrieved from PPCM-treated VEC or VLC using a MOI of 0.01 for 12 h (Fig. 4). No EBOV glycoprotein staining was seen either in PPCM-treated infected cultures at day 7 post infection when looking at 5 fields of view at low magnification (data not shown). Altogether, these data suggest that PPCM has an antiviral activity against EBOV in the context of a sexually-transmitted infection, which is consistent with our previous study using VK2/E6E7 cells (Escaffre et al., 2019).

### 3.4. VLC inflammatory response to EBOV infection and beneficial effect of PPCM

**Fig. 5.**
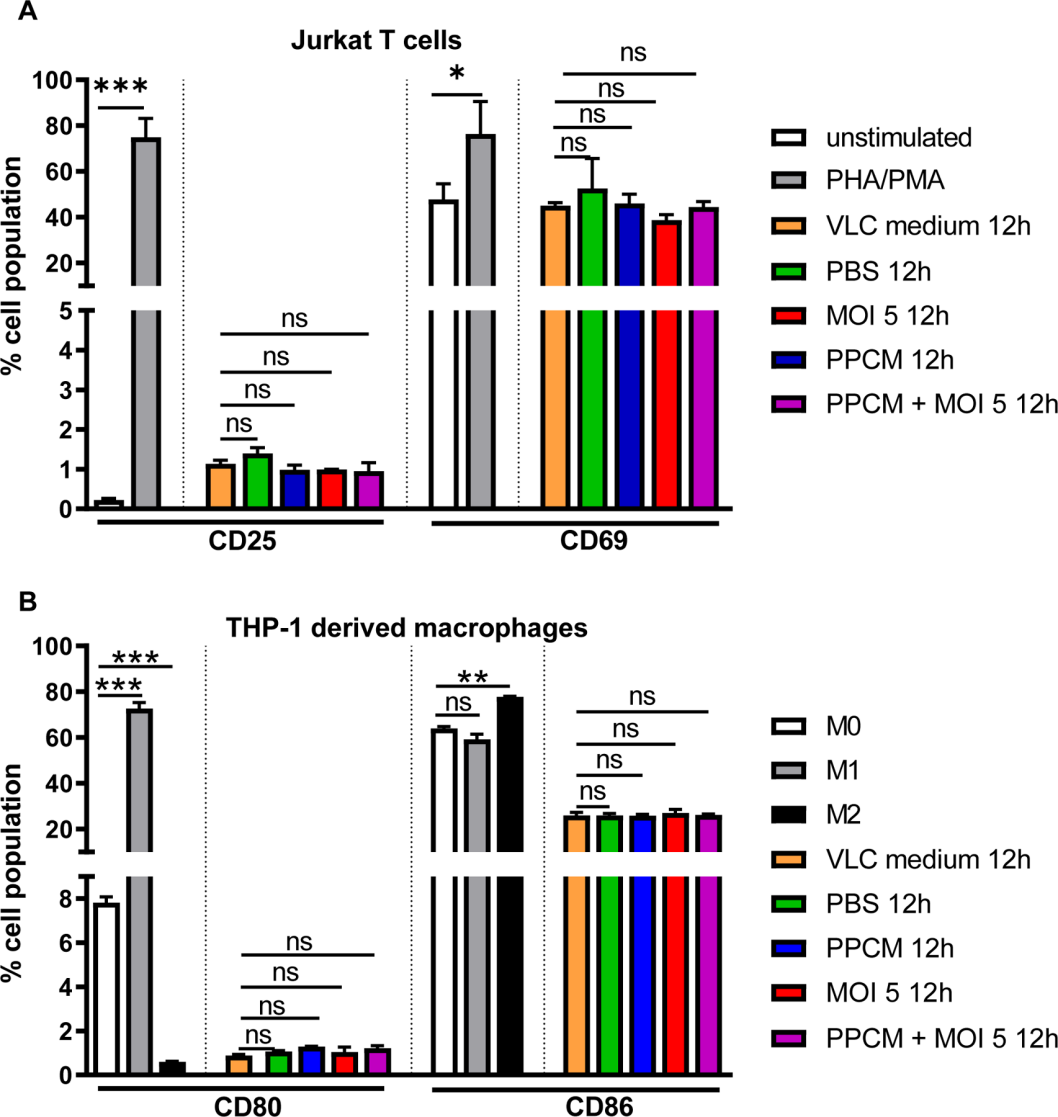
Assessment of VLC inflammatory response to EBOV through measurement of surface markers on immune cells by flow cytometry. Quantification of CD25/CD69 and CD80/CD86 expression in respectively Human CD4 T lymphocytes (Jurkat cell line) (**A**) or resting (M0) macrophage-derived monocytes (THP-1 cell line) (**B**) following 48 h incubation in VLC samples from day 6 and 7 time point. Percentage of cell population expressing a given marker is obtained by averaging data of 2 biological replicates. Error bars represent standard deviations. ns for non-significant, * p < 0.05, ** p < 0.01, *** p < 0.001 (ANOVA-Tukey’s multiple-comparison test).

**Fig. 6.**
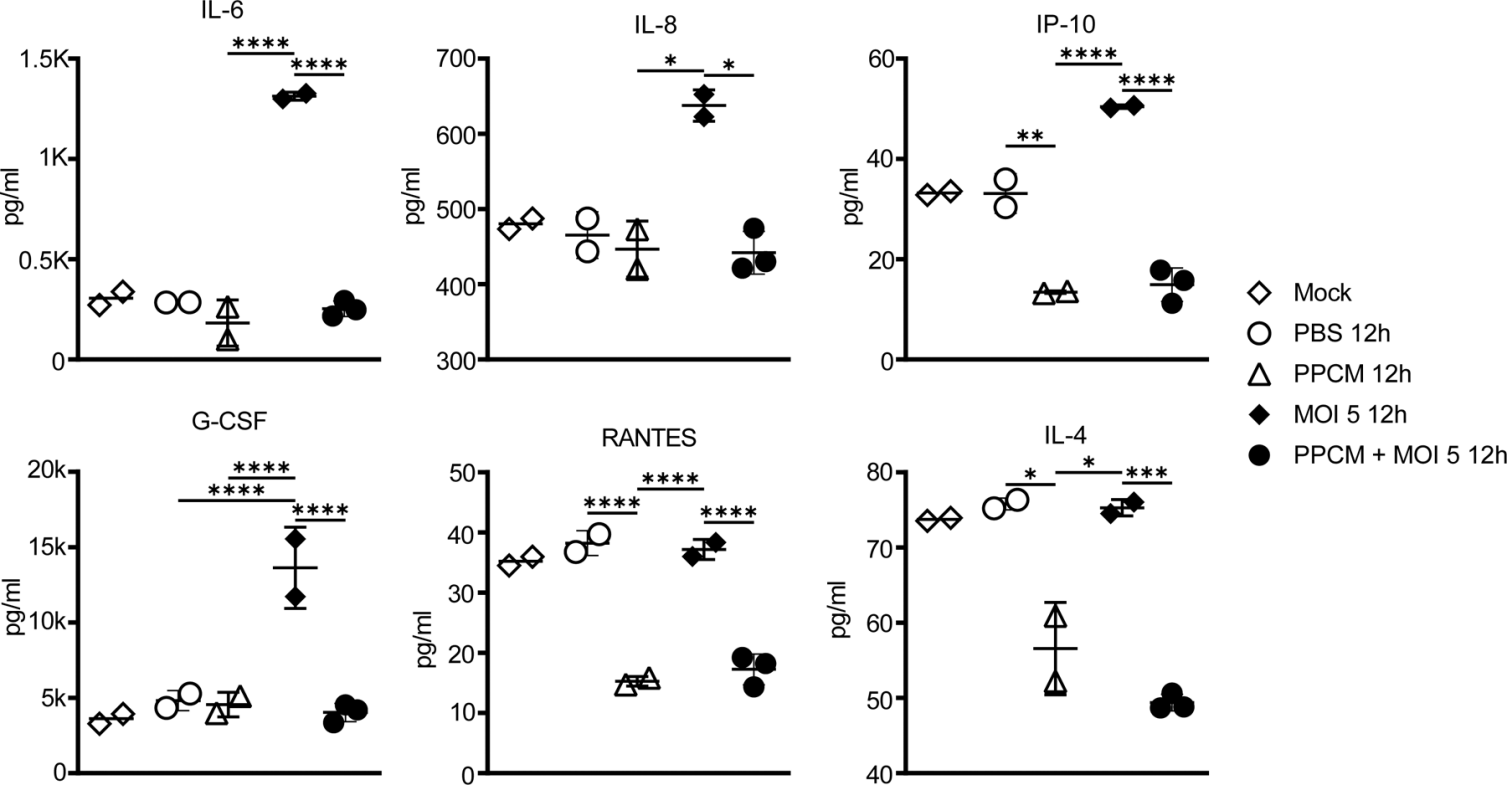
Quantification of inflammatory markers from EBOV-infected VLC. Proinflammatory cytokines and chemokines secreted from the basal side of noninfected (white symbols) or high MOI-infected (black symbols) VLC at day 2 post infection. Results are expressed as the average of 2–3 biological replicates and error bars represent standard deviations. * p < 0.05, ** p < 0.01 and *** p < 0.001, (ANOVA-Tukey’s multiple-comparison test).

An exacerbated systemic release of inflammatory mediators constitutes one of the hallmark of EVD (Feldmann et al., 2013). Therefore, samples collected from the basal side of cultures were analyzed for their ability to further amplify the immune response. However, only VLC samples were used as both models allowed for detectable (MOI 5) and comparable levels of infectious virus with or without PPCM. Also, VLC is potentially more complete with dendritic cells whose infection is relevant in EBOV pathogenesis (Lubaki et al., 2013). First, the ability of VLC samples at activating immune cells was assessed through changes in the level of expression of cell surface markers. This is justified by the fact that immune cell migration was previously seen in EBOV antigen-positive vaginal tissues in macaques (Liu et al., 2022). Human CD4 T lymphocytes (Jurkat cell line) as well as macrophage-derived monocytes (M0 derived from THP-1 cell line) were incubated for 48 h in pooled VLC samples from day 6 and 7 time points. Changes in expression levels of CD25/CD69 (Yiemwattana et al., 2012; Sasagawa et al., 2006; Weiss et al., 1984; Manger et al., 1986; Cheng et al., 2015; Shatrova et al., 2015) and CD80/CD86 (Ambarus et al., 2012; Jaguin et al., 2013) were then quantified in CD4 T cells or in M0, respectively. While PMA/PHA treatment control increased expression of CD25 and CD69 in CD4 T cells (p < 0.05), VLC control medium and insert-derived samples only hardly and similarly increased expression of the former surface marker (Supplementary Fig. 3, Fig. 5A). Polarization of resting macrophages (M0) into classically (M1) and alternatively (M2)-activated macrophages was translated, as expected, by an increased expression of respectively CD80 (p < 0.001) or CD86 (p < 0.01), when compared to the reciprocals in M0. However, VLC medium and insert-derived samples conversely equally decreased the basal level of both markers of M0 (Supplementary Fig. 4, Fig. 5B). As the lack of lymphocyte and macrophage activation could stem from either absence of their cognate ligands, interference of VLC medium, not using primary cells, or not targeting other soluble and cell surface markers, all samples were then directly analyzed for their content in proinflammatory analytes. Among 27 different mediators tested, only the secretion levels of interleukin (IL)-6, IL-8, IP-10, and Granulocyte colony stimulating factor (G-CSF) were significantly increased by EBOV infection at day 2 (p < 0.05, or p < 0.0001) post infection when a high infective dose was used (Fig. 6). However, concentration levels of these analytes were comparable between samples originating from mock-, PBS-, and 4% PPCM-treated inserts as well as 4% PPCM-treated infected inserts (p > 0.05), in line with our previous study using VK2/E6E7 cells (Escaffre et al., 2019). Besides, levels of RANTES and IL-4 in all samples from 4% PPCM-treated inserts were significantly lower compared to those from mock-, PBS-, and virus-treated inserts (p < 0.05; p < 0.001, or p < 0.0001). Note that a comparable profile for all these cytokines was observed at day 4 post-infection (data not shown). Altogether, these data suggest that 4% PPCM does not cause inflammation of the vaginal epithelium and that no inflammation will take place should the tissue be exposed to EBOV following PPCM treatment.

## 4. Discussion

Establishing EBOV sexual transmission is challenging because presence of virus in vaginal secretions could be due to either systemic spread, in line with reports from EBOV studies in animal models (Cooper et al., 2018; Liu et al., 2022; Perry et al., 2018) and at least three case studies (Rodriguez et al., 1995; Liu et al., 2019; WHO, 2015), or as a result of intercourse with local infection. The latter is more difficult to demonstrate as vaginal swabs are typically taken in convalescent patients or after disease onset (>4 days) and viremia (Rodriguez et al., 1995; Thorson et al., 2016; WHO, 2015; Rogstad and Tunbridge, 2015). Therefore, the conclusion of sexual transmission in women mostly falls back on whether the sequence of the virus found in the semen of an EBOV survivor matches with the counterpart isolated from the blood of the partner (Mate et al., 2015; Subissi et al., 2018; Christie et al., 2015). Consistent with these reports, we previously showed in vitro that human vaginal and cervical epithelial cells were permissive to EBOV (Escaffre et al., 2019). Besides, the former cells mounted a robust inflammatory response to infection, which could be significantly reduced along with virus replication, when using the broad-spectrum polyphenylene carboxymethylene (PPCM) microbicide. There are currently no newer in vitro data or studies performed in animal models demonstrating that route of transmission, and filovirus infection of the human reproductive system and systemic spread from there is still poorly understood. Here, a follow up study of EBOV infection of the human vaginal epithelium was done using three-dimensional air-liquid interface primary models that are more biologically relevant than VK2/E6E7 submerged cultures to provide realistic data regarding the early steps of EBOV infection in the context of a sexual transmission, and the ability of PPCM vaginal microbicide at preventing it.

The human vaginal epithelium is a mucosal surface that is at the frontline for interaction with pathogens. This epithelium consists of multicellular stratified squamous epithelial cell layers sitting on a lamina propria (Anderson et al., 2014). Specifically, it is made of a basal layer (*stratum basale*), a suprabasal layer, a superficial layer, and a final layer of flattened cornified cells (*stratum corneum*), whose latter was reported to only be partially present in the VEC model (Ayehunie et al., 2015), which is overall also in line with our findings in both VEC and VLC. VEC is a well-accepted model to mimic the human vaginal epithelium in vitro due to its similar architecture and its hormone responsiveness (Ayehunie et al., 2015), which made it attractive to study sexually transmitted diseases (Garcia et al., 2019; Pandit et al., 2019) or to test microbicides, spermicides and feminine hygiene products

(Ayehunie et al., 2006, 2011, 2015, 2018; Garcia et al., 2019; Pandit et al., 2019; Kaur et al., 2011). VLC is a more recent model built upon VEC that includes dendritic cells that yet we could not detect when targeting key markers for this cell type (Pena-Cruz et al., 2018; Wu et al., 2018; Zhao et al., 2003) suggesting that our batch did not have this type of cells. However, their presence is not excluded and other markers such as CD123, or CD303 could be used instead, but this will require further investigations. Altogether, our data suggest that VEC and VLC are biologically relevant to evaluate EBOV infection and for drug testing. The increase of permeability over time in all inserts was unexpected and caused by apparent tissue retraction from the outer rim of the inserts. This was not previously reported with VEC (Ayehunie et al., 2006, 2011, 2015, 2018; Garcia et al., 2019; Pandit et al., 2019), and while resolving this issue is beyond the scope of this study, it could originate from improper concentrations of estrogen and progesterone that will modulate tissue differentiation and thickness (Ayehunie et al., 2015).

Consistent with confirmed cases of sexual transmission (Mate et al., 2015; Subissi et al., 2018; Christie et al., 2015) and our initial in vitro study using undifferentiated cells (Escaffre et al., 2019), both models could be readily infected throughout the entire architecture suggesting that the virus can invade the vaginal connective tissue. The fact that viral load shed by either model following a low infective dose challenge was below the limit of detection while virus antigen was sometimes found in the most apical layers could reflect a delay or inefficient infection. Note that the minimal viral load required to a successful vaginal infection is still unknown but is believed to be much lower (Bausch et al., 2007) compared to a MOI of 5. Together with the fact that EBOV can invade this epithelium in animals through viremia (Cooper et al., 2018; Liu et al., 2022), our data strongly support the fact that EBOV can be efficiently transmitted through the vaginal route and also highlights the advantage of using complex and differentiated models over two-dimensional submerged monocultures.

The low toxicity of PPCM was previously demonstrated in many cell types, and 5–500 μg/ml showed effectiveness against EBOV regardless of the infective dose used and treatment type (Escaffre et al., 2019). Considering that VLC and VEC recapitulate the vaginal epithelium architecture, as opposed to VK2/E6E7 cells, PPCM was used here at a higher dose of 40 mg/ml which was well tolerated over a 12 h exposure. This is in line with the safety profile of this compound that was determined during vaginal toxicity and irritation studies using the same dose and where no weight loss, signs of disease, or significant vaginal lesions were seen in rabbits after 10 days of repeated daily exposure (Zaneveld et al., 2002). Here, the beneficial effect of using PPCM was demonstrated from the high infective dose data as pretreatment significantly decreased to an undetectable level (lower than 7 pfu/ml) the quantity of infectious virus produced between day 2 and 7 from a titer at least 2.9 log_10_ higher, which is also consistent with our previous findings (Escaffre et al., 2019). Note that the measurable virus titer at 12 h post infection in PPCM-treated samples corresponds to the first wash after removing the inoculum and likely reflects dilution of PPCM to a suboptimal concentration in the subsequent plaque assay rather than the actual infectivity of the samples. When extrapolating these results to the kinetic data from a 500-times lower infective dose, which is more relevant in case of a natural infection (Bausch et al., 2007), our data suggest that PPCM can abolish infection of the vaginal tissue. However, it is currently unknown how effective at preventing infection of the cervix and uterus PPCM can be, as seen in macaques (Liu et al., 2022; Perry et al., 2018), since application of any microbicides beyond the vaginal canal could be challenging.

VEC model can activate several immunity-related canonical pathways and secrete inflammatory markers following hormonal and microbicide treatments (Ayehunie et al., 2006, 2015), which makes this model along with VLC interesting to study sexually transmitted infections. Here, the immune response of the vaginal epithelium to infection and PPCM treatment was first explored by assessing how VLC samples affect the expression level of activation markers from circulating or neighboring immune cells. Specifically, T lymphocytes and macrophages were used as they are respectively indirect and direct EBOV targets (Bradfute et al., 2010; Bray and Geisbert, 2005; Younan et al., 2018), and both types, along with virus antigen, have been found infiltrated in the lamina propria of the vaginal epithelium of EBOV-infected macaques (Liu et al., 2022). Also, the surface markers chosen in this study have been shown to be induced in various immune cells in the context of EBOV infection (Bosio et al., 2004; Geisbert et al., 2003; Kotliar et al., 2020; Madelain et al., 2016; Wagstaffe et al., 2020; Warfield et al., 2003). However, the fact that none of the VLC samples could activate T cells suggests that there was no to too little of specific ligands and appropriate co-stimulatory molecules to do so (Williams and Bevan, 2007). Alternatively, detecting intra-cellular expression of IL-2, IL-4, IFN-γ, and TNF-α is also well-accepted as proof of activation but this will require further investigations. Polarization of resting macrophages into activated macrophages with respectively a pro and anti-inflammatory phenotype (Ambarus et al., 2012; Jaguin et al., 2013; Tedesco et al., 2018; Wang et al., 2019) was translated, as expected and seen in our study, by distinct changes in CD80 and CD86 expression markers. However, the fact that all M0 incubated with VLC samples had little CD80 expression, similar to M2 profile, along with significantly less CD86 compared to the reciprocal in M0, M1, M2, suggest interference or toxicity of VLC medium that could mask a potential activation. While these data were inconclusive, we believe they will be helpful to other groups interested in studying VLC responses to any stimulus should an immune cell chemotaxis assay or similar be developed.

As an alternative method, VLC response to infection was directly measured from VLC samples by multiplex immunoassay. We previously showed that EBOV infection of a VK2/E6E7 monolayer cells significantly increased secretion of chemokines (Eotaxin, IL-8, IP-10, MIP-1β, and RANTES) and cytokines (IFN-β, IFN-γ, IFN-λ1, IFN-λ2, IL-6, IL-7, IL-9, IL-11, and TNF-α) (Escaffre et al., 2019). In line with the present study and although a smaller set of analytes was investigated (59 vs. 27), IL-8, IP-10, and IL-6 levels were again increased following infection. G-CSF cytokine level was also elevated by infection in VLC but not in VK2/E6E7 cells that are yet of the same origin. Conversely, no increase in IFN-γ was detected using VLC altogether suggesting that VLC immune response is more channeled than that of the monolayer cells and is likely due to the differentiation process. This reflects another benefit of using complex and differentiated primary models over two-dimensional monocultures. Nevertheless, a significant increase of the above analytes suggests inflammation through activation of the NF-κB pathway (Martinez et al., 2007), and is in line with the fact that acute hemorrhagic vaginitis was seen in EBOV-infected macaques (Liu et al., 2022). Consistent with our previous study (Escaffre et al., 2019), PPCM prevented virus-induced inflammation as the secretion levels of IL-6, IL-8, IP-10, G-CSF, RANTES, and IL-4 were either comparable or lower than those in Mock-infected cells. Interestingly, increase of IL-6 and IL-8 have been shown to reflect cell toxicity (Fichorova et al., 2004) and could contribute to vaginal epithelial barrier disruption (Schlievert et al., 2022) suggesting that PPCM can also reduce inflammation-induced tissue permeability.

In conclusion, we characterized two similar vaginal epithelium models cultured at air-liquid interface and tested their susceptibility to EBOV. While data from EBOV replication in VEC and VLC versus VK2/E6E7 cells are of limited novelty we show in the present study that EBOV infected all epithelial layers and some cells in the lamina propria which is indicative of virus penetration and suggestive of effective sexual transmission through that route. EBOV infection also resulted in an increased secretion of inflammatory markers, with some analytes being different from those identified in VK2/E6E7 cells and is overall consistent with vaginitis. We finally confirmed PPCM antiviral and anti-inflammatory effect when added prior exposure as it is intended to use. Our previous and current vitro data using VK2/E6E7, VEC, and VLC models support the need to further study sexual transmission of EBOV and warrant in vivo studies as this route of infection is still reported on a yearly basis (April 2022; CDC, 2020; CDC, 2021; CDC, 2017; CDC, 2018). The effectiveness of PPCM should also be further assessed in vitro and in vivo against all filoviruses.

## Supplementary Material

2

1

3

4

## Data Availability

Data will be made available on request.
