# Supplementary figures and images for: Characterization of an air-liquid interface primary human vaginal epithelium to study Ebola virus infection and testing of antivirals
Source: Antiviral Res. Author manuscript; Available in PMC 2023 Jun 22. (PMC10286122; doi:10.1016/j.antiviral.2023.105551)

Supplementary figure 2

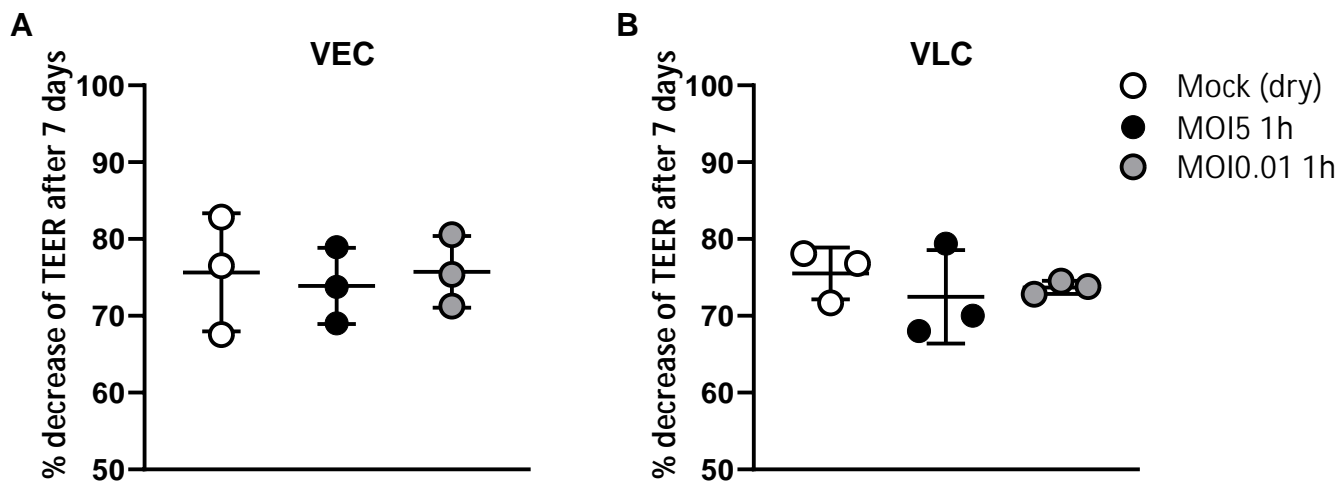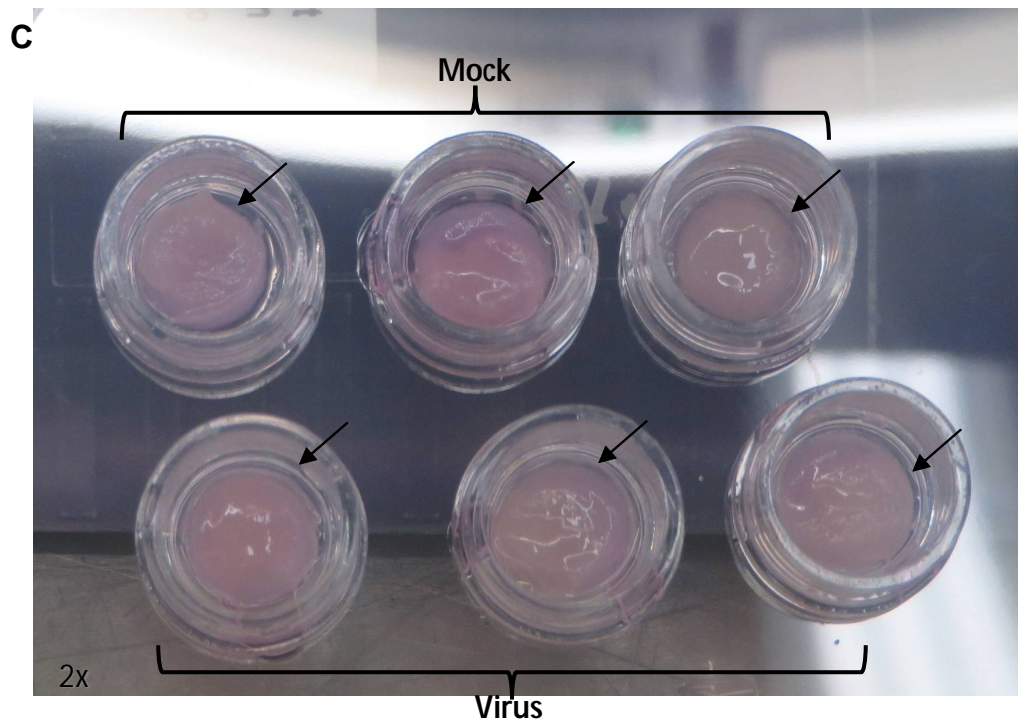

Supplement: 2 [file NIHMS1905620-supplement-2.pdf]

Supplementary figure 1

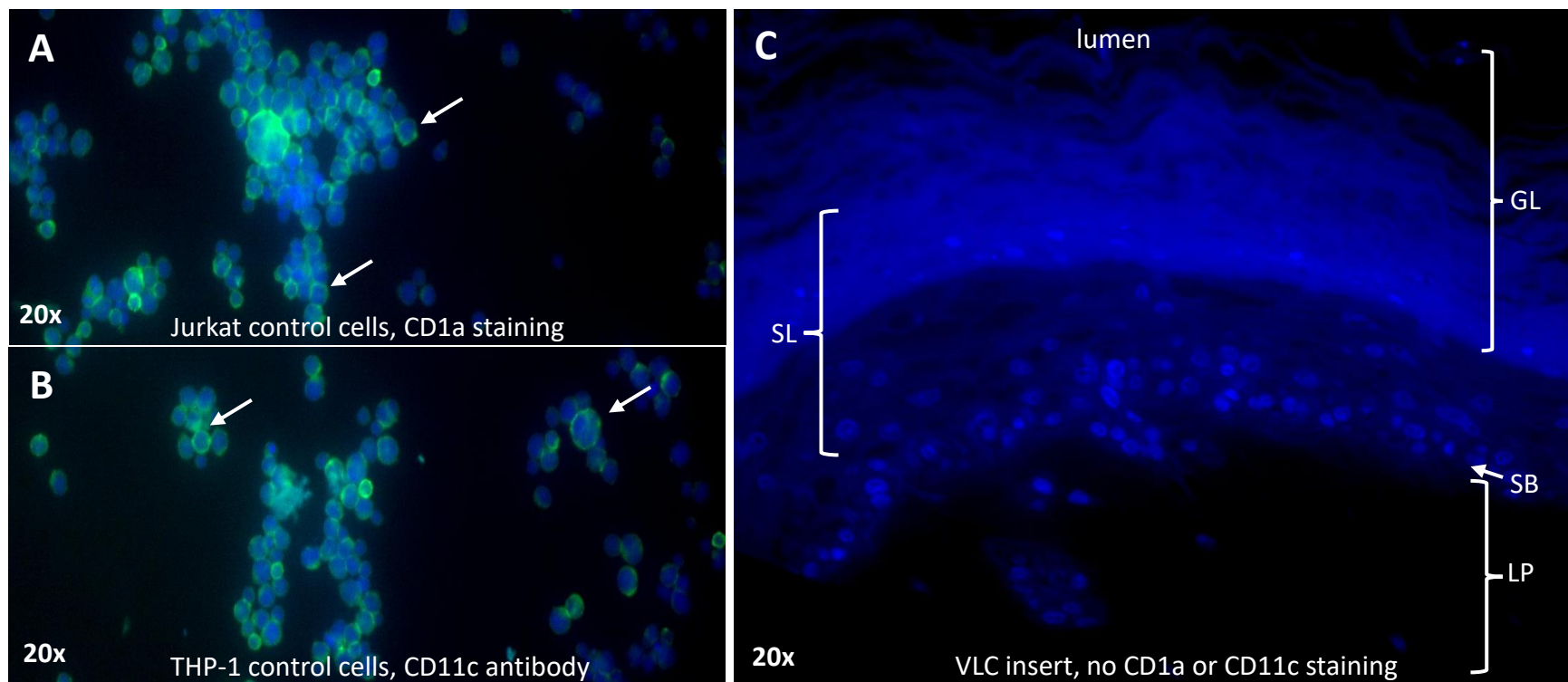

Supplement: 1 [file NIHMS1905620-supplement-1.pdf]

Supplementary figure 3

Jurkat T lymphocytes

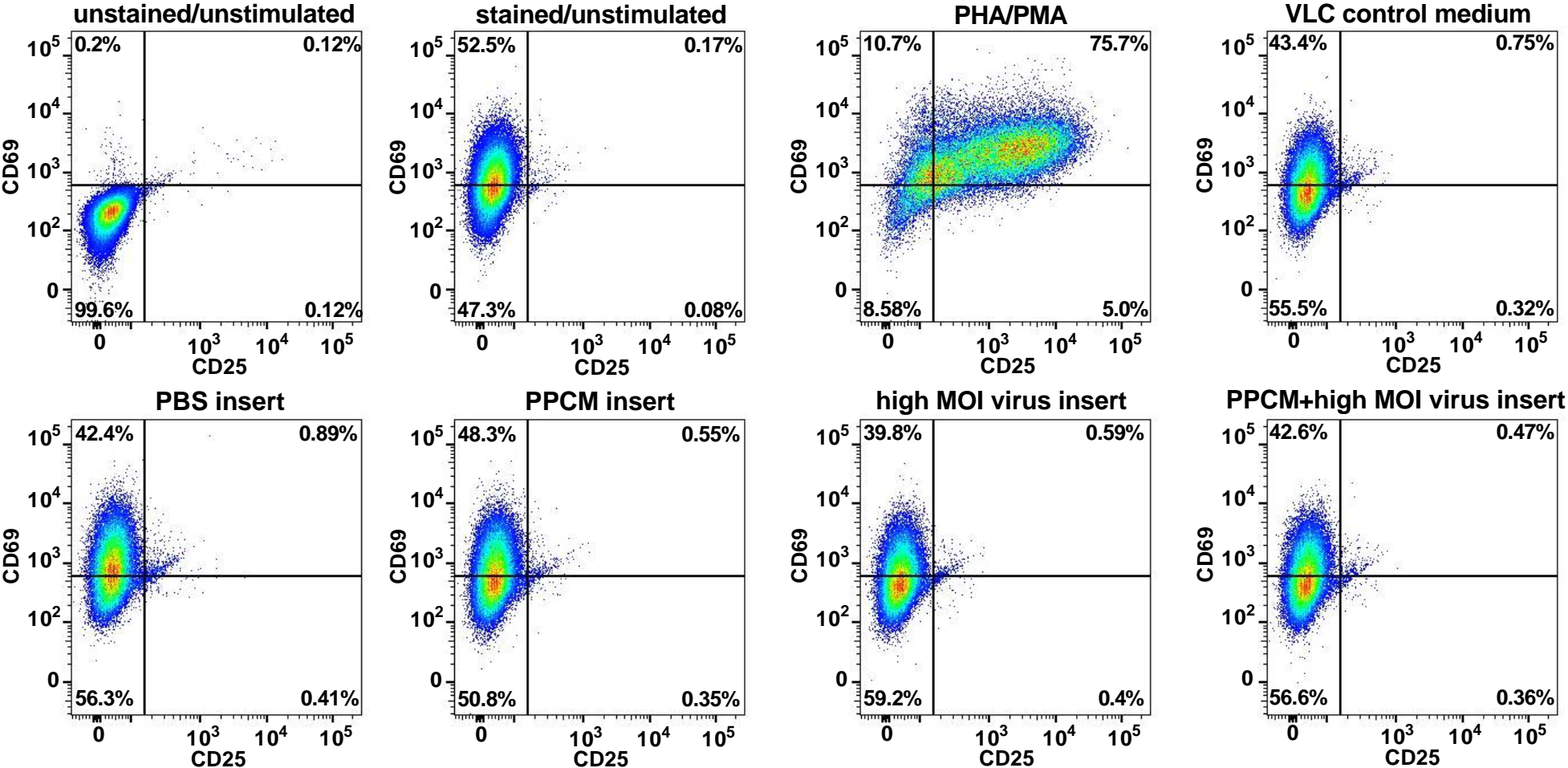

Supplement: 3 [file NIHMS1905620-supplement-3.pdf]

Supplementary figure 4

THP-1-derived macrophages

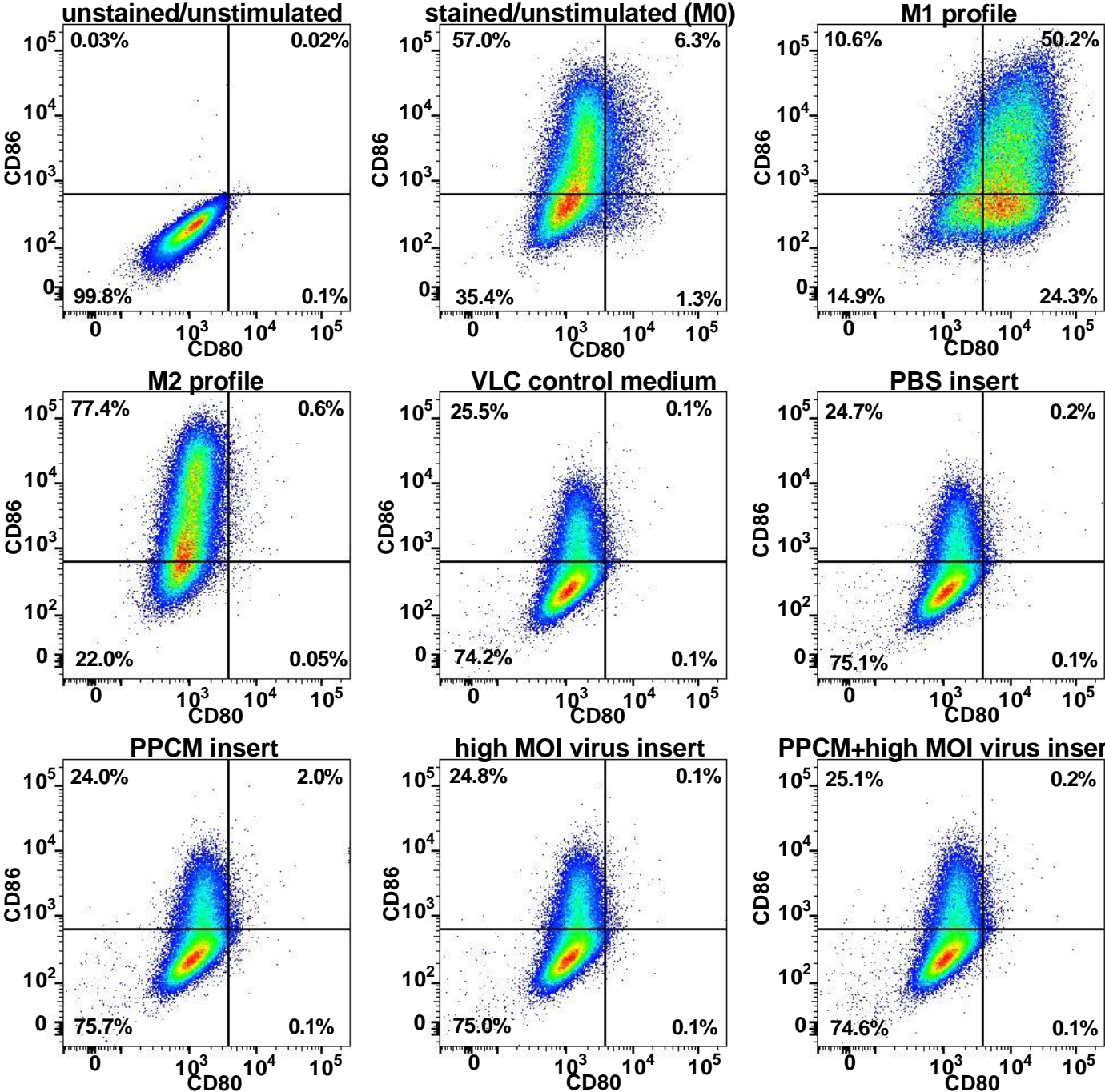

Supplement: 4 [file NIHMS1905620-supplement-4.pdf]
